# Integrating Sequence Capture and Restriction Site-Associated DNA Sequencing to Resolve Recent Radiations of Pelagic Seabirds

**DOI:** 10.1093/sysbio/syaa101

**Published:** 2021-02-06

**Authors:** Joan Ferrer Obiol, Helen F James, R Terry Chesser, Vincent Bretagnolle, Jacob González-Solís, Julio Rozas, Marta Riutort, Andreanna J Welch

**Affiliations:** 1 Departament de Genètica, Microbiologia i Estadística, Facultat de Biologia, Universitat de Barcelona, Barcelona, Catalonia, Spain; 2 Institut de Recerca de la Biodiversitat (IRBio), Barcelona, Catalonia, Spain; 3 Department of Vertebrate Zoology, National Museum of Natural History, Smithsonian Institution, Washington, DC, USA; 4 U.S. Geological Survey, Patuxent Wildlife Research Center, Laurel, MD, USA; 5 Centre d’Études Biologiques de Chizé, CNRS & La Rochelle Université, 79360, Villiers en Bois, France; 6 Departament de Biologia Evolutiva, Ecologia i Ciències Ambientals, Facultat de Biologia, Universitat de Barcelona, Barcelona, Catalonia, Spain; 7 Department of Biosciences, Durham University, Durham, UK

## Abstract

The diversification of modern birds has been shaped by a number of radiations. Rapid diversification events make reconstructing the evolutionary relationships among taxa challenging due to the convoluted effects of incomplete lineage sorting (ILS) and introgression. Phylogenomic data sets have the potential to detect patterns of phylogenetic incongruence, and to address their causes. However, the footprints of ILS and introgression on sequence data can vary between different phylogenomic markers at different phylogenetic scales depending on factors such as their evolutionary rates or their selection pressures. We show that combining phylogenomic markers that evolve at different rates, such as paired-end double-digest restriction site-associated DNA (PE-ddRAD) and ultraconserved elements (UCEs), allows a comprehensive exploration of the causes of phylogenetic discordance associated with short internodes at different timescales. We used thousands of UCE and PE-ddRAD markers to produce the first well-resolved phylogeny of shearwaters, a group of medium-sized pelagic seabirds that are among the most phylogenetically controversial and endangered bird groups. We found that phylogenomic conflict was mainly derived from high levels of ILS due to rapid speciation events. We also documented a case of introgression, despite the high philopatry of shearwaters to their breeding sites, which typically limits gene flow. We integrated state-of-the-art concatenated and coalescent-based approaches to expand on previous comparisons of UCE and RAD-Seq data sets for phylogenetics, divergence time estimation, and inference of introgression, and we propose a strategy to optimize RAD-Seq data for phylogenetic analyses. Our results highlight the usefulness of combining phylogenomic markers evolving at different rates to understand the causes of phylogenetic discordance at different timescales. [Aves; incomplete lineage sorting; introgression; PE-ddRAD-Seq; phylogenomics; radiations; shearwaters; UCEs.]

Understanding the phylogenetic relationships among species is paramount in biology and provides a framework for understanding evolutionary processes such as temporal and biogeographical patterns of diversification. Radiations are one of the major challenges in reconstructing evolutionary history and examples of recalcitrant clades are widespread across the Tree of Life (Song et al. 2012; Wagner et al. 2013; Jarvis et al. 2014; Pease et al. 2016). Such rapid diversification events commonly generate patterns of phylogenetic incongruence that hinder the understanding of major evolutionary processes.

Two prevalent processes contribute to incongruence. One of them is incomplete lineage sorting (ILS), which occurs when gene lineages coalesce into their common ancestor prior to the speciation events (Maddison 1997). Under ILS, retention and stochastic sorting of ancestral polymorphisms may result in misleading resolution of relationships among species. ILS is particularly prevalent at short internal branches in species trees and especially when effective population sizes (Ne) are large relative to the time between divergences (Pamilo and Nei 1988; Rosenberg and Nordborg 2002). The second process is introgression, the incorporation (usually via hybridization and backcrossing) of alleles from one species into the gene pool of a second species (Anderson 1949). Introgression plays an important role in the process of diversification (Abbott et al. 2016) and is especially important in adaptive radiations (The Heliconius Genome Consortium et al. 2012; Malinsky et al. 2018). Distinguishing ILS from introgression remains a major challenge in phylogenomics. Recently, several methodological approaches have been developed that simultaneously account for both ILS and gene flow when reconstructing the evolutionary history of a clade (Cruaud et al. 2014; Wen et al. 2018). The footprints of these processes on sequence data can, however, vary between different phylogenomic markers at different phylogenetic scales depending on factors such as their evolutionary rates or the type of selection that they experience (Martin and Jiggins 2017; Knowles et al. 2018).

The increasing availability of tractable phylogenomic data has certainly helped in resolving many contentious relationships in the Tree of Life (Rokas et al. 2003; Jarvis et al. 2014; Hughes et al. 2018). Genome-wide data can detect patterns of gene tree discordance and can be used to investigate the causes of phylogenetic incongruence in rapid diversification events (Arcila et al. 2017). Restriction site-associated DNA sequencing (RAD-Seq; Miller et al. 2007) and sequence capture of ultraconserved elements (UCEs; Faircloth et al. 2012), are two of the most widely used methods for shallow phylogenomics. RAD-Seq and related genotyping-by-sequencing approaches (e.g., ddRAD-Seq, Peterson et al. 2012; GBS, Elshire et al. 2011) have been primarily used for studying polymorphism in population genomics analyses, although they have also been successfully used for phylogeographic and interspecific phylogenetic studies of a wide variety of organisms (Emerson et al. 2010; Hohenlohe et al. 2010; Rubin et al. 2012). Conversely, UCEs were developed for studying deep evolutionary timescales but their flanking regions are variable enough to be informative at shallow evolutionary timescales (Faircloth et al. 2012; Smith et al. 2014). These two methods have recovered concordant phylogenetic relationships when using large enough data sets (Leaché et al. 2015; Harvey et al. 2016; Manthey et al. 2016; Collins and Hrbek 2018). RAD-Seq and UCEs have also been compared in terms of divergence time estimation using fossil-calibrated molecular-clock models and have been shown to accurately estimate divergence times at recent timescales (Collins and Hrbek 2018). Integrating these two approaches may provide a powerful tool for disentangling the roles of ILS and introgression in rapid diversification events; however, empirical studies to test this assumption are generally lacking.

Modern birds provide many case studies, as their diversification has been characterized by a succession of rapid radiations, posing a significant challenge in resolving the phylogenetic relationships of several avian clades (Jarvis et al. 2014; Oliveros et al. 2019). Hybridization can be common at the species level (Mallet 2005) and birds show relatively high levels, with 16.4% of the species having been documented to hybridize in nature (Ottenburghs et al. 2015).

Evolutionary relationships of tube-nosed seabirds (order Procellariiformes) are unresolved at many phylogenetic levels (Penhallurick et al. 2004; Rheindt and Austin 2005). Many species of Procellariiformes are endangered (Croxall et al. 2012) and shearwaters are amongst the most vulnerable groups; 55% of shearwater species are listed as threatened by the IUCN Red List of Threatened Species. Shearwaters form a monophyletic group of medium-sized pelagic seabirds (family Procellariidae) consisting of three genera. Understanding shearwater diversification and biogeographic patterns can provide important insights into their biology, and ultimately assist in providing species delimitations or evolutionarily significant units vital for their conservation (Purvis et al. 2005).

Resolving the evolutionary relationships among shearwaters has long been challenging. Osteological and morphological analyses have generated many conflicting hypotheses (Kuroda 1954), likely due in part to their slowly evolving osteological characters and remarkable similarities in plumage coloration, which also make their identification in the wild challenging (Gil-Velasco et al. 2015). Mitochondrial DNA (mtDNA) analysis (Austin 1996; Heidrich et al. 1998; Austin et al. 2004; Pyle et al. 2011) revealed further conflicts and suggested polytomies may exist among and within the three shearwater genera (particularly within major biogeographic groups in the genus *Puffinus*). A recent analysis of the major clades of Procellariiformes using only UCE loci (Estandia 2019) recovered a generally well resolved topology, though maximum likelihood and species tree estimation yielded lower support and conflicting topologies for the split among the shearwaters, suggesting a rapid radiation where ILS and/or historical introgression may have occurred. Hybridization has been documented in several species of Procellariiformes, including shearwaters (Genovart et al. 2012; Booth Jones et al. 2017; Masello et al. 2019) despite strong philopatry to breeding colonies and, in most cases, a lack of overlap between breeding areas of closely related species. Thus, shearwaters demonstrate the challenges typical of many other taxonomic groups, where rapid diversification and introgression hinder attempts to confidently resolve their evolutionary history, and provide a good case study for how combining genomic data sets can aid in clarifying relationships at recalcitrant nodes.

Here, we generate the first phylogenomic data sets for shearwaters. We use paired-end ddRADSeq (PE-ddRAD) and UCE data sets to explore the role of ILS and introgression as causes of phylogenetic discordance during rapid diversification. We adopt a thorough and integrative approach, comparing and combining the two data sets, and applying state-of-the-art concatenated and coalescent-based approaches using shearwaters as a case study. We expand previous comparisons of UCE and RAD-Seq data sets for phylogenetics, divergence time estimation and inference of introgression, and we propose a strategy to optimize RAD-Seq data for phylogenetic analyses. Our approach allows us to completely resolve the phylogenetic relationships of shearwaters. We detect high levels of gene tree discordance associated with short internodes, mainly caused by ILS, and report a potential case of historical introgression. We show that our integrative approach provides a good framework for exploring the processes underlying phylogenetic incongruence during rapid diversification events.

## Materials and Methods

### Sampling and Sequence Data Generation

We obtained blood ($n = 45$), high-quality tissue ($n = 19$) or dry tissue ($n = 3$) samples for 30 taxa representing 26 of the 30 recognized species of shearwaters (Carboneras and Bonan 2019) (Supplementary Table S1 available on dryad at http://dx.doi.org/10.5061/dryad.d51c5b00t). We also sampled three species of Procellariiformes as outgroups: *Fulmarus glacialis*, a species from the same family as the shearwaters (Procellariidae); and two more distantly related outgroups, *Thalassarche chlororhynchos* (Diomedeidae) and *Oceanites oceanicus* (Oceanitidae). Species that could not be included (*Puffinus heinrothi*, *Puffinus bannermani*, *Puffinus persicus*, and *Puffinus subalaris*) are mostly very localized or critically endangered.

We used the Qiagen DNeasy Blood and Tissue Kit to extract genomic DNA according to the manufacturer’s instructions (Qiagen GmbH, Hilden, Germany). For dry tissue samples, we extracted DNA using the Dabney et al. (2013) ancient DNA extraction protocol. We used a Qubit Fluorometer (Life Technologies) to quantify and standardize DNA concentrations of all samples. PE-ddRAD data for the outgroups were retrieved from whole genome assemblies available from the Bird 10K project (Feng et al. 2020; see description of the *in silico* digestion protocol in the Data Assembly—PE-ddRAD-Seq data set section below).

Library preparation, capture enrichment and sequencing of the UCEs was conducted by RAPiD Genomics, LLC (Gainesville, FL, USA). Briefly, genomic DNA was fragmented, and sequencing libraries were prepared. Indexed samples were subjected to PCR for seven cycles prior to pooling. UCE loci were captured using the Tetrapods UCE-5Kv1 probe set (available at ultraconserved.org) as described by Faircloth et al. (2012), and PCR was conducted for 11 cycles to amplify the enriched library. Sequencing was performed on two lanes of an Illumina HiSeq 3000 platform using 100 bp paired-end (PE) sequencing.

Library preparation of PE-ddRAD loci was performed by the Genomic Sequencing and Analysis Facility, University of Texas at Austin, following the Peterson et al. (2012) protocol. Samples were digested using an uncommon cutter *EcoRI* and a common cutter *MspI* in a single reaction. After digestion, modified P1 Illumina adapters containing 5 bp unique barcodes and P2 Illumina adapters were ligated onto the fragments and individually barcoded samples were pooled. Barcodes differed by at least two base pairs (based on a Hamming distance metric) to reduce the chance of errors caused by inaccurate barcode assignment. Pooled libraries were size selected (between 150 and 300 bp after accounting for adapter length) using a Pippin Prep size fractionator (Sage Science, Beverly, Ma). Libraries were amplified in a final PCR step for 10 PCR cycles in six pools differing by their Illumina index, prior to sequencing on a single lane of an Illumina HiSeq4000 platform using 150 bp PE sequencing. The combination of unique barcodes and Illumina indexes allowed the multiplexing of all samples into one sequencing lane.

### Data Assembly

**Figure 1:**
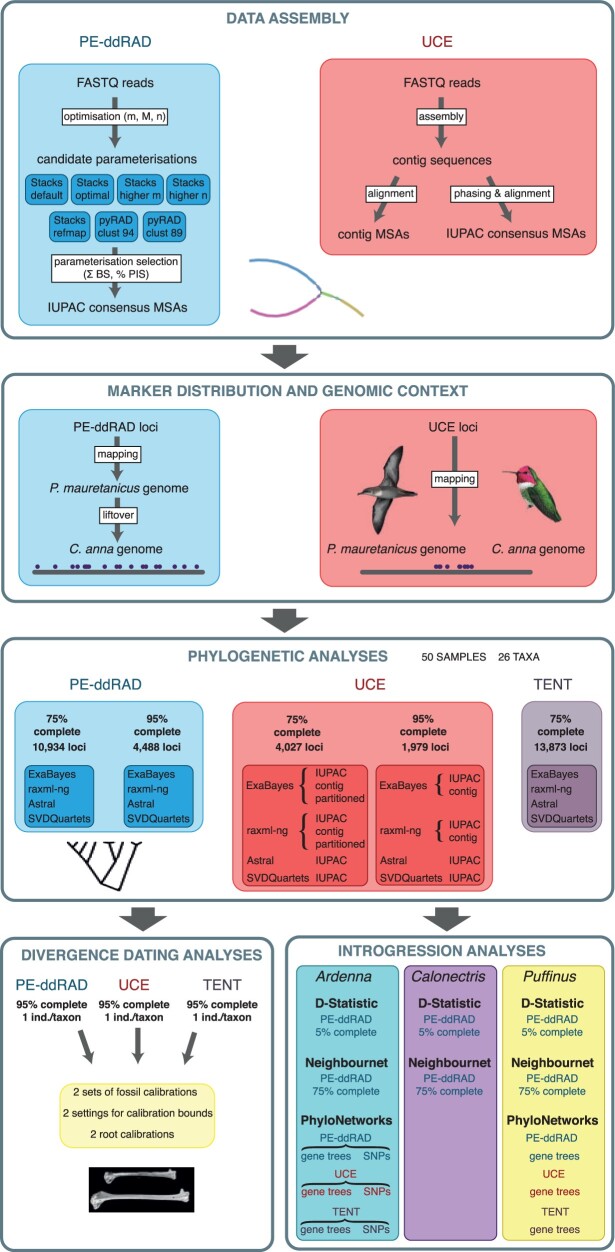
Graphic outline of the analyses carried out in this study. Balearic shearwater illustration by Martí Franch and Anna’s hummingbird illustration reproduced with permission from Lynx Edicions.

*UCE* *data set.*—Raw reads were quality-filtered and cleaned of adapter contamination with Trimmomatic v0.36 (Bolger et al. 2014), and were assembled into contigs using Trinity v2.0.6 (Grabherr et al. 2011) as implemented in the PHYLUCE pipeline (Faircloth 2016). To identify contigs representing UCE loci, we mapped all assembled contigs to the probes’ reference sequences (uce-5k-probes.fasta) and discarded those contigs not matching any probe, matching more than one probe or matching probes that matched multiple contigs, using the PHYLUCE match_contigs_to_probes.py script. The remaining UCE sequences were aligned using MAFFT v.7.130b (Katoh and Standley 2013) and internally trimmed using Gblocks v.0.91b (Castresana 2000). To retrieve polymorphism information lost when collapsing multiple reads into a single contig sequence, we used the phasing protocol described by Andermann et al. (2018) to obtain International Union of Pure and Applied Chemistry (IUPAC) consensus sequence alignments comparable to the PE-ddRAD alignments. Finally, we assembled data sets containing UCE loci that were present in at least 75% and 95% of the taxa using contig alignments (UCE 75 contig and UCE 95 contig) and using IUPAC consensus sequence alignments (UCE 75 IUPAC and UCE 95 IUPAC) (Fig. 1).

#### PE-ddRAD-Seq data set

We quality-filtered and demultiplexed reads using process_radtags in Stacks v2.41 (Rochette et al. 2019). To obtain PE-ddRAD data sets optimized for phylogenomic analyses, we performed a two-step approach to assembling RAD loci (Fig. 1). Here we provide an overview of this approach, which is described in detail in the Supplementary Information available on dryad. First, we used a method for optimizing *de novo* assembly of loci using Stacks (Paris et al. 2017; Rochette and Catchen 2017) which consists of varying each of the two key parameters (M: within-individual distance parameter, and n: between-individual distance parameter; Catchen et al. 2011) separately using denovo_map.pl, and selecting values of M and n under which the tendency of new polymorphic loci when increasing M or n becomes linear. We also assessed the frequency of putative sequencing errors in the data by selecting the value of m (stack-depth parameter) where the proportion of singletons in the data set stabilized (Harvey et al. 2016). Secondly, we used two metrics to assess the effect of different pipelines and different parameterizations when assembling PE-ddRAD data sets: the number of parsimony informative sites (PIS) per locus relative to total locus length (pPIS); and the sum of bootstrap branch support (BS) obtained from maximum likelihood analyses (ML). For the second step, we used seven parameterizations from two assembly pipelines, Stacks and pyRAD 3.0.66 (Eaton 2014) (Fig. 1 and Supplementary Table S2 available on Dryad). For all analyses, we extracted PE-ddRAD loci *in silico* from the outgroups using the python script Digital_RADs.py (DaCosta and Sorenson 2014).

We used PE-ddRAD IUPAC consensus sequence alignments from the selected parameterization (Stacks higher m) to assemble two data sets containing loci present in at least 75% and 95% of the samples (PE-ddRAD 75 and PE-ddRAD 95) for downstream analyses.

#### Total evidence data set

To reduce the effect of data type on phylogenetic inference, we combined UCE and PE-ddRAD IUPAC consensus sequence alignments. We obtained a total evidence data set with those UCE and PE-ddRAD loci present in at least 75% of the taxa (TENT 75). PE-ddRAD and UCE markers may overlap. To avoid including a sequence twice in our analyses, we used Blastn (Altschul et al. 1997) to map representative PE-ddRAD loci sequences to the UCE loci. We removed any PE-ddRAD loci that mapped to a UCE locus prior to concatenation.

### Marker Distribution and Genomic Context

Because the genomic distribution of phylogenomic markers can assist with understanding their informativeness across phylogenetic scales, we compared the distributions and genomic context of PE-ddRAD and UCE loci. We used to map both sets of loci to the Balearic Shearwater (BaSh; *Puffinus mauretanicus*) draft genome assembly (Cuevas-Caballé et al. 2019) and to the most closely related chromosome-level genome assembly, the Anna’s Hummingbird (AnHu; *Calypte anna*; (Korlach et al. 2017), which diverged between 62.7 and 71.1 Ma (Jarvis et al. 2015). Due to the large divergence time between shearwaters and AnHu, we also mapped the PE-ddRAD markers to the AnHu genome using a liftover approach (see Supplementary Information available on dryad). To determine the level of clustering of UCE and PE-ddRAD loci, and to determine their degree of association with protein-coding genes, we applied a permutation procedure (Bioconductor package regioneR; Gel et al. 2016), using 5000 permutations for each analysis (see Supplementary Information available on dryad).

### Phylogenetic Analyses

We used unpartitioned UCE and PE-ddRAD concatenated data sets (UCE IUPAC 75, UCE IUPAC 95, PE-ddRAD 75, and PE-ddRAD 95) and a total evidence concatenated data set (TENT 75) partitioned by data type (UCE and PE-ddRAD) to estimate Bayesian and maximum-likelihood phylogenies using the MPI version of ExaBayes v.1.5 (Aberer et al. 2014) and raxml-ng v.0.6.0 (Kozlov et al. 2019), respectively. Additionally, we estimated PE-ddRAD ML trees including the ingroup taxon *Puffinus assimilis haurakiensis*, for which no UCE data were available due to sampling constraints. For each data set, we ran two independent ExaBayes runs with four coupled chains for 1,000,000 generations. We assessed runs for stationarity in Tracer v.1.7 (Rambaut et al. 2018) by checking for effective sample sizes $>$ 300 for all model parameters. We created a consensus tree from the two independent runs using the consense programme from the ExaBayes package (burnin: 25%). We ran raxml-ng with the GTR$+$G substitution model to conduct 50 ML tree searches using 25 random and 25 parsimony-based starting trees. Following the best tree search, we generated 500 nonparametric bootstrap replicates. We checked for convergence post-hoc using the—bsconverge command in raxml-ng with a cutoff value of 0.03; we computed branch support values and mapped the values onto the best-scoring ML tree using the raxml-ng—support command.

We performed Bayesian and ML concatenated analyses using UCE unpartitioned contig alignments (UCE 75 contig and UCE 95 contig) to test the accuracy of the phylogenetic estimation of this approach (Fig. 1). Concatenated unpartitioned analyses can converge to a tree other than the species tree (Roch and Steel 2015) and produce highly supported but incorrect nodes in the tree (Kainer and Lanfear 2015). To verify that our data sets were not affected by these issues, we also performed analyses of UCE partitioned IUPAC consensus alignments (75% complete). We used the Sliding-Window Site Characteristics (SWSC-EN) method described in Tagliacollo and Lanfear (2018) and PartitionFinder 2 (Lanfear et al. 2017) to partition the data, which yielded 131 partitions. These analyses are explained in detail in the Supplementary Information available on dryad.

To account for coalescent stochasticity among individual loci, we inferred species trees with IUPAC UCE, PE-ddRAD, and TENT data sets using two multispecies coalescent methods: the quartet-based method SVDQuartets (Chifman and Kubatko 2014), and the summary method ASTRAL-III (Zhang et al. 2018). SVDQuartets can handle both unlinked single nucleotide polymorphisms (SNPs) and multilocus data. To allow a better comparison with analyses based on concatenation we ran SVDQuartets on the multi-locus sequence alignments. Analyses were run in PAUP* v.4 (Swofford 2002) evaluating all possible quartets. For each matrix, we conducted 100 bootstrap replicates, and results were summarized in a 50% majority-rule consensus tree. For ASTRAL-III, we used RAxML v.8 (Stamatakis 2014) to estimate gene trees for each PE-ddRAD and UCE locus in the IUPAC 75% complete data sets. We ran 500 rapid bootstrap replicates for each individual gene followed by a thorough ML search and we estimated species trees from the best-scoring ML gene trees and bootstrap replicates using ASTRAL-III. Two analyses were run for each data set: one using the original gene trees and one using gene trees with very low support branches (BS $<$ 10) contracted as this procedure can improve tree accuracy (Zhang et al. 2018). Branch support values were inferred using local posterior probabilities (PP; Sayyari and Mirarab 2016). To avoid the negative impacts of fragmentary gene sequences on gene tree and species tree reconstruction (Sayyari et al. 2017), we removed three samples with mean missing data values per locus higher than 5% (*A. carneipes* 1, *A. grisea* 1 and *C. diomedea* 2) for ASTRAL-III analyses of UCE data sets. We annotated ASTRAL-III trees with local quartet supports for the main topology, and the first and second alternatives (ASTRAL-III option -t 2) to further investigate regions of the species tree that are potentially in the anomaly zone (Degnan and Rosenberg 2006). Finally, we performed a polytomy test (Sayyari and Mirarab 2018) to evaluate whether hard polytomies could be rejected at short internodes.

All phylogenetic analyses were conducted using only *F. glacialis* as an outgroup after checking that preliminary analyses using all outgroups yielded the same results (Supplementary Fig. S11 available on Dryad). This decision was made to avoid long-branch attraction (Felsenstein 1978) and systematic error due to highly divergent outgroup taxa (Graham et al. 2002).

### Divergence Time Estimation

In divergence date estimation analyses, it has been common practice to reduce data set size by selecting clock-like genes (Smith et al. 2018). Nonetheless, we decided to perform the analyses using the three 95% complete data sets (UCE, PE-ddRAD, and TENT), because recent research has shown that divergence time analyses using complete phylogenomic data sets consistently show less variance in divergence times than estimates using subsets of clock-like genes (McGowen et al. 2019; Oliveros et al. 2019). We acknowledge that divergence time estimation methods based on concatenation may lead to branch-length bias and potentially misleading age estimates, particularly for younger divergence times (McCormack et al. 2011; Angelis and dos Reis 2015). However, as our objective was not necessarily to calculate accurate estimates but rather to compare estimates based on different phylogenomic markers, we decided to use a concatenation-based method that allowed us to use complete phylogenomic data sets.

Divergence time analyses were performed using MCMCTree v.4.9 from the PAML package (Yang 2007). MCMCTree allows Bayesian divergence time inference of phylogenomic data sets (dos Reis and Yang 2011) by implementing approximate likelihood calculation. We pruned the topology of our ExaBayes TENT 75% tree and the TENT 75 data set so that they contained one individual per taxon to be used as input for divergence dating analyses, retaining the most complete individual. To decide the best-fitting clock model, we used the stepping-stones method (Xie et al. 2011) as implemented in the mcmc3r R package (dos Reis et al. 2018) to calculate marginal likelihoods for relaxed-clock models using the computationally expensive exact likelihood method because the approximate likelihood method cannot be used for marginal likelihood calculation (dos Reis et al. 2018). Thus, we carried out model selection on smaller subsets of the data suitable for exact likelihood calculation (two randomly selected subsets of 60 UCE loci and two randomly selected subsets of 120 PE-ddRAD loci, averaging 20,000 bp each). The marginal likelihoods were then used to calculate posterior probabilities for the strict, independent and autocorrelated rate models (ST, IR, and AR, respectively).

We performed divergence dating analyses using: 1) two different fossil calibration strategies using 4 (A) and 3 (B) node calibrations (root calibration included in both strategies); 2) maximum and minimum soft bounds or only minimum bounds; and 3) two different maximum ages for the root calibration. For all analyses, maximum and minimum bounds were set for the root calibration. We provide detailed justifications for the four fossil calibrations used in the study (Marsh 1870; Miller 1961; Olson and Rasmussen 2001; Olson 2010) in the Supplementary Information available on Dryad.

We followed the two-step procedure outlined in dos Reis and Yang (2011) to infer divergence times using approximate likelihood calculation. For each analysis, we ran two independent Markov chain Monte Carlo (MCMC) chains, collecting 10,000 samples after a burn-in of 5000 and a sample frequency of 500. We assessed likelihood convergence and parameters by examining trace plots in Tracer and checking that the estimated sample size (ESS) for each parameter was not smaller than 300, and by comparing results between independent runs. We also ran MCMCs with no data to generate joint prior distributions. Finally, we generated infinite-sites plots to assess how uncertainty in time estimates differed between analysis of the three data sets.

### GC-biased Gene Conversion

GC-biased gene conversion (gBGC) is known to strongly affect several features of avian genomes (Nabholz et al. 2011; Weber et al. 2014). To investigate potential signatures of GC-biased gene conversion (gBGC) in base composition and substitution rates in shearwaters, SNP data from the PE-ddRAD 75 data set were output in VCF format using the populations program in Stacks. For biallelic variant sites, we computed reference and minor allele frequencies using VCFtools v0.1.15 (Danecek et al. 2011). We assigned variant sites into one of the following mutation categories: strong-to-strong (S-to-S), strong-to-weak (S-to-W), weak-to-strong (W-to-S), and weak-to-weak (W-to-W), where C and G are strong bases (3 hydrogen bonds) and A and T are weak bases (2 hydrogen bonds). Although we recognize that the ancestral allele cannot be assigned with certainty (Keightley and Jackson 2018), due to the lack of outgroup sequences, we polarized variant sites based on their frequencies, with the minor allele being considered as derived. To investigate the role of gBGC on minor allele frequencies, we compared the distributions of minor allele frequencies between the different mutation categories, and we explored the change in their prevalence depending on the local GC content.

To test expectations that gBGC is more effective in species with large population sizes and/or species with smaller body mass (Romiguier et al. 2010; Weber et al. 2014), we compared the number of breeding pairs and the average body mass to the overall proportion of W-to-S mutations per species. The number of breeding pairs per species and average body masses were retrieved from the Handbook of the Birds of the World (Carboneras and Bonan 2019).

### Introgression Analyses

#### Split Networks

To better visualize patterns of genealogical discordance and potential areas of reticulate evolution, we computed phylogenetic networks for each genus using the Neighbour-Net approach (Bryant and Moulton 2004). Analyses were implemented in SplitsTree version 5 (Huson and Bryant 2006), using default parameters.

#### Patterson’s D-statistic (ABBA-BABA Test)

To further explore whether tree discordances are due to past introgression or other forms of model misspecification, we quantified the Patterson’s D-statistic (Green et al. 2010; Patterson et al. 2012) for all species quartets compatible with the time-calibrated topology. Calculations were performed using Dsuite Dtrios (Malinsky et al. 2021) with a PE-ddRAD-derived SNP data set that included loci with data in at least 5 taxa (ddRAD_min5, 295,779 SNPs). We performed analyses for the three genera separately. In each analysis, the sister species to the rest of the genus was used as the outgroup (i.e., *P. nativitatis*, *C. leucomelas*, and *A. bulleri* and *A. pacifica*). Block-jackknife resampling was used to evaluate significant deviations from zero in Patterson’s D-statistic ($P< 0.001$). Significant results were validated using different outgroups and also using the pyRAD implementation of the statistic.

For those cases with a significant Patterson’s D-statistic, we extracted SNPs with strong signatures of introgression (i.e., SNPs with ABBA configuration and fixed within species, hereafter “ABBA SNPs”) to evaluate alternative potential causes of these signatures, such as shared ancestral variation, mutational hotspots resulting in convergent mutations, gBGC, and levels of genetic variation (see Supplementary Information available on Dryad).

#### Phylogenetic Network Analyses

We reconstructed phylogenetic networks using the maximum pseudolikelihood method implemented in SNaQ (Solís-Lemus and Ané 2016; Solís-Lemus et al. 2017). This method accommodates ILS and gene flow under the multispecies network coalescent model (MSNC). We ran phylogenetic networks for each of the data sets (PE-ddRAD, UCE, and TENT) and independently for *Puffinus* and *Ardenna*, to reduce computation time and to improve the accuracy of the inferred networks (Solís-Lemus and Ané 2016; Solís-Lemus et al. 2017). In both cases, we used the time-calibrated topology, pruned to only contain taxa in the genus under study, as a starting topology. For each data set, we conducted 10 independent runs with random seeds of SNaQ to infer the optimal coalescent tree with no hybridization edges (h0). We then performed network searches from 1 to 4 (1 to 2 for *Ardenna* data sets) hybridization edges providing, in each case, the optimal network with hmax-1 hybridization edges. The preferred number of hybridizations was selected based on the analysis of the slope of a plot of log-pseudolikelihood against the number of hybridisations (Solís-Lemus and Ané 2016; Solís-Lemus et al. 2017). We expected a sharp improvement until the number of hybridization edges reached the best value. We did not evaluate more than four (or two in the case of *Ardenna*) hybridization edges because of the lack of change in the slope heuristic.

To assess whether the MSC adequately explained gene-tree discordance to our coalescent trees with no hybridization edges (h0), we used the Tree Incongruence Checking (TICR R; Stenz et al. 2015), using the phylolm R package (Tung and Ané 2014). A chi-squared test was used to compare observed concordance factors (CF) with expected CF calculated from the h0 species trees under the MSC. We further looked for taxa that did not fit the tree model with ILS retrieving the outlier 4-taxon sets.

Because searches for an optimal network produced inconsistent results in independent runs and in different data sets, we focused on evaluating candidate reticulation events based on the results of the Patterson’s D-statistics. We optimized the pseudodeviance of candidate networks using the topologyMaxQPseudolik! function and we visualized the estimated inheritance probabilities ($\gamma$).

## Results

### Data Assembly

We recovered a mean number of PE-ddRAD and UCE reads of 1,273,325 (SD $=$ 876,938) and 1,851,330 (SD $=$ 517,189) per sample, respectively (Supplementary Table S1 available on Dryad). We assembled an average of 25,716 PE-ddRAD tags per sample; the alignment lengths per locus ranged from 140 to 239 bp with a median of 198 bp (SD $= 25.5$). UCE Trinity contigs ranged from 213 to 1565 bp with a median length of 551 bp (SD $= 126.6$) and an average recovery of 83.6%.

Correlations between different UCE summary statistics showed that low sequencing yield not only resulted in low sequencing coverage but also in a lower number of assembled loci, which were shorter on average, and in a lower percentage of sequencing on target (see Supplementary Information and Supplementary Fig. S1 available on Dryad).

Our results for the *de novo* optimization of PE-ddRAD are described in detail in the Supplementary Information available on Dryad. Briefly, the tendency of new polymorphic loci when increasing M or n parameters in Stacks, became linear at M $= 5$ and n $= 8$ (Supplementary Fig. S2 available on Dryad) and the proportion of singletons in the data set stabilized at m $= 7$ (Supplementary Fig. S3 available on Dryad). For the second optimization step, different parameterizations had a minor effect on the pPIS with the exception of the default parameters in Stacks that yielded loci with a much lower number of PIS. With similar parameterizations, Stacks yielded approximately twice the number of loci than pyRAD for each level of missing data, but pyRAD loci had a slightly higher pPIS (Supplementary Fig. S4a and Table S2 available on Dryad). Phylogenetic analyses using the different data sets and levels of missing data resulted in overall highly resolved and congruent phylogenies. However, the general trend was a slight increase in resolution when reducing missing data from a maximum of 35% to a maximum of 25% and thereafter, a slight decrease when reducing it to a maximum of 5% (Supplementary Fig. S4b available on Dryad). Phylogenetic analyses using data sets with a higher amount of missing data (35% and 25%) tended to yield higher bootstrap supports on recent splits, whereas analyses using data sets with a low level of missing data (5%) yielded higher bootstrap supports on more ancient splits (Supplementary Fig. S5 available on Dryad). pyRAD data sets with a maximum of 25% missing data yielded the highest overall resolution but Stacks higher m data sets were the most consistent across different levels of missing data. Thus, we selected the latter parameterization for downstream analyses.

For the same taxon coverage, PE-ddRAD concatenated alignments were both longer (i.e., 2,156,937 bp for the PE-ddRAD 75 matrix vs. 1,732,076 bp for the UCE 75 matrix) and contained more than double the number of loci than UCE alignments (Table 1). The number of PIS per locus was very similar between PE-ddRAD and UCE alignments despite the much shorter length of PE-ddRAD loci.

**Table 1.** Characteristics of assembled data sets used in phylogenetic analyses

	Taxon	Number	Median	Number	Median
	coverage	of	locus	of	PIS per
Method	(%)	loci	length (SD)	PIS	locus (SD)
PE-ddRAD	75	10,934	198 (25.51)	85,070	7 (4.19)
PE-ddRAD	95	4488	205 (19.47)	34,505	7 (4.18)
UCE	75	4027	452 (121.06)	31,664	6 (7.13)
UCE	95	1979	484 (109.75)	13,900	6 (6.45)

### Marker Distribution and Genomic Context

**Figure 2:**
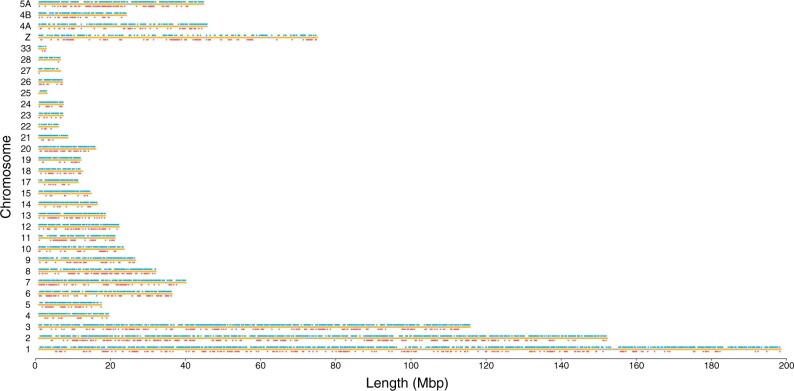
Genomic distribution of PE-ddRAD (blue) and ultraconserved elements (red) when mapped to the chromosome-level genome assembly for *Calypte anna*. Chromosomes are represented as gray lines and the yellow spots on the chromosomes represent the location of protein-coding genes.

Using Blastn, 97.7% of the UCE and 95.4% of the PE-ddRAD loci successfully mapped to the *P. mauretanicus* draft genome assembly. When mapped to the more distant *C. anna* chromosome-level genome assembly, an even higher percentage of UCE loci successfully mapped (99.4%) which contrasted with the low percentage of successfully mapped PE-ddRAD loci (30.6%). Using the liftover approach, we managed to improve the percentage of successfully mapped PE-ddRAD loci to the *C. anna* genome assembly to 77.5%. UCE loci had a higher level of clustering than PE-ddRAD loci (median distance between the closest UCE loci $= 16.2$ kbp, median distance between the closest PE-ddRAD loci $= 51.3$ kbp; Fig. 2, Supplementary Figs. S6, S7, and S8 available on Dryad). PE-ddRAD loci were closer to protein-coding genes (51.4 $\pm $ 94.0 kbp) than UCEs (73.8 $\pm $ 104.8 kbp) (Supplementary Figs. S9 and S10 available on Dryad).

### Phylogenomic Analyses

**Figure 3:**
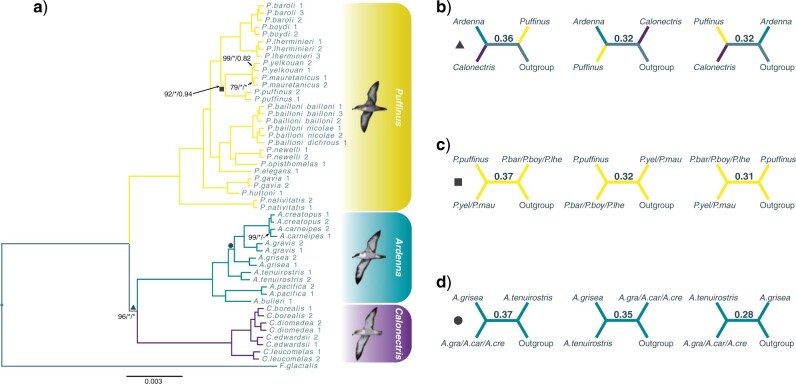
a) Phylogram of the concatenated bayesian tree inferred in ExaBayes using the total evidence (TENT) data set: a 75% complete matrix of ultraconserved elements (UCE) and paired-end double-digest Restriction site-Associated DNA (PE-ddRAD) loci. All nodes have 100% bootstrap support values (BS; raxml-ng) and 1.0 posterior probabilities (PP; ExaBayes and ASTRAL-III) unless labeled otherwise. Labels correspond to raxml-ng BS/ExaBayes PP/ASTRAL-III PP with asterisks indicating full support and hyphens indicating nodes not recovered. The three nodes represented by different shapes resulted in incongruence in analyses using different genomic markers, levels of missing data and/or phylogenetic methods. Quartet supports for these nodes from the ASTRAL-III analysis using the same data set are shown in b) to d) for the main and the two alternative quartet topologies. Illustrations by Martí Franch represent the three shearwater genera.

Phylogenetic analyses recovered largely the same well-resolved tree topology across different genomic markers, levels of missing data, and phylogenetic methods. The phylogenomic tree resulting from the TENT 75 ExaBayes analysis is shown in Figure 3a and results of all phylogenetic analyses performed in this study are detailed in Supplementary Table S3 available on Dryad. All analyses supported the monophyly of the three recognized genera of shearwaters: *Ardenna, Calonectris*, and *Puffinus*. *Ardenna* and *Calonectris* were sister genera and together were the sister lineage to the species-rich *Puffinus*. Within *Puffinus*, we recovered *P. nativitatis* as the sister taxon to the remaining *Puffinus* species, which formed five strongly supported and biogeographically defined clades: a clade from New Zealand and Australian waters (*P. gavia* and *P. huttoni*); a Subantarctic and New Zealand clade (*P. elegans* and *P. assimilis haurakiensis*); a North Pacific clade (*P. newelli* and *P. opisthomelas*); a Tropical Indian and South Pacific clade (*P. bailloni*); and a Caribbean, North Atlantic and Mediterranean clade (*P. puffinus*, *P. mauretanicus*, *P. yelkouan*, *P. lherminieri*, *P. boydi*, and *P. baroli*) (Fig. 3a and Supplementary Fig. S12 available on Dryad).

**Figure 4:**
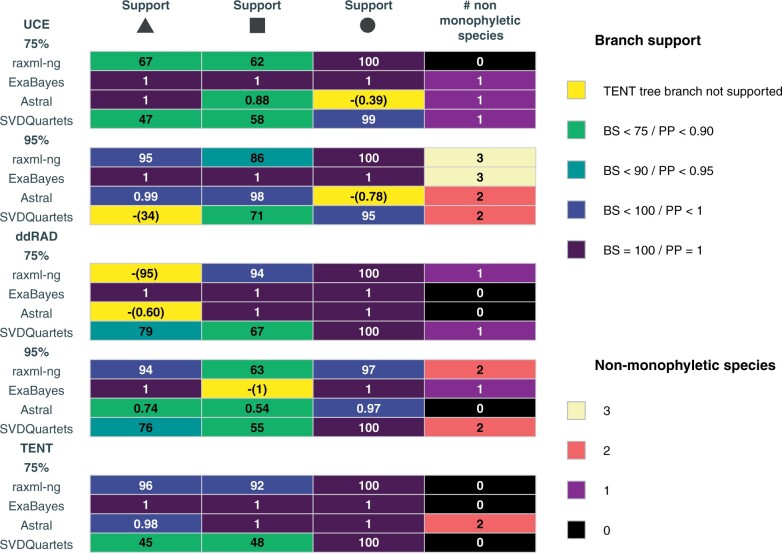
Heatmap showing support for the challenging branches highlighted in Figure 3 and the number of non-monophyletic species recovered in analyses using different genomic markers, levels of missing data and phylogenetic methods. Every row corresponds to a different analysis with a particular data set and every column corresponds to one of the challenging branches highlighted in Fig. 3, with the exception of the last column that corresponds to the number of non-monophyletic species recovered. Values are shown in each tile and tiles are colored corresponding to the legend. In analyses where a particular branch was not recovered (indicated with a dash), the support for the alternative arrangement is shown in parentheses.

Only three phylogenetic relationships, all localized to short internodes, were challenging to resolve due to discordances between analyses using different genomic markers, levels of missing data, or phylogenetic methods (Fig. 3b–d). The first discordance was the relationship among the three shearwater genera (triangle). All analyses using the TENT data set and most analyses using the UCE and the PE-ddRAD data sets recovered *Calonectris* as sister to *Ardenna* (Fig. 4). However, raxml-ng and ASTRAL-III trees based on the PE-ddRAD 75 data set and the SVDQuartets tree based on the UCE 95 data set recovered the alternative topology (*Ardenna* as sister to *Puffinus*). A polytomy test based on local quartet supports (Fig. 3b) using the TENT data set marginally ruled out that this branch should be replaced by a polytomy ($P = 0.0324$). Under a true polytomy, we would expect local quartet supports for the three alternative topologies to be equal to 1/3. This test was also significant when using only UCE data but failed to discard the likelihood of a polytomy when using PE-ddRAD data (Table 2). The second discordance was the relationship between the three North Atlantic lineages of *Puffinus* (Fig. 3c). In this case, all phylogenetic analyses recovered *P. puffinus* as the sister species to the Mediterranean *P. mauretanicus* and *P. yelkouan*, with the exception of the PE-ddRAD 95 ExaBayes tree, which recovered *P. puffinus* as the sister to *P. lherminieri*, *P. baroli*, and *P. boydi*. Despite consistency in the recovered relationships for this case, only the polytomy tests based on the PE-ddRAD 75 and TENT 75 data sets were able to reject the null hypothesis that the branch should be replaced by a polytomy. The last case was the relationship between the two all dark species of *Ardenna* (*A. tenuirostris* and *A. grisea*) (Fig. 3d). The only analyses that recovered *A. grisea* and *A. tenuirostris* as sister species used coalescent-based methods and UCE data sets (Fig. 4), although it should be noted that ASTRAL-III analyses using UCEs were performed with only one *A. grisea* individual due to missing data filtering (see Phylogenomic Analyses in Materials and Methods section). Interestingly, all tests rejected a polytomy although UCE data sets supported one topology and PE-ddRAD data sets another (Table 2), showing different phylogenetic signals between both types of markers. It is also noteworthy that the monophyly of the most recently diverged taxa *P. mauretanicus*–*P. yelkouan* and *A. creatopus*–*A. carneipes* was not supported in several phylogenetic analyses (Supplementary Table S3 available on Dryad).

**Table 2.** ASTRAL-III quartet supports for the main and the two alternative quartet topologies and polytomy test $P$-values for the challenging branches highlighted in Fig. 3. The null hypothesis is polytomy and $P$-values $<$ 0.05 reject a real polytomy. When the alternative topology was recovered (indicated with a dash), polytomy test $P$-values are shown in parentheses. Note that only the TENT data set is able to reject real polytomies at the three challenging branches

		UCE		ddRAD		TENT
Branch	Value	75%	95%	75%	95%	75%
$\blacktriangle$	Polytomy test $P$-value	0.008	0.010	-(0.261)	0.257	0.032
	QS: (*Ardenna*, *Calonectris*)	0.39	0.4	0.34	0.35	0.36
	QS: (*Calonectris*, *Puffinus*)	0.32	0.32	0.31	0.31	0.32
	QS: (*Ardenna*, *Puffinus*)	0.29	0.28	0.35	0.34	0.32
$\blacksquare$	Polytomy test $P$-value	0.190	0.034	0.003	0.802	0.001
	QS: (*P. puffinus*, (*P. mauretanicus*, *P. yelkouan*))	0.37	0.4	0.37	0.34	0.37
	QS: (*P. puffinus*, (*P. lherminieri*, *P. boydi*, *P. baroli*))	0.32	0.31	0.32	0.33	0.32
	QS: ((*P. mauretanicus*, *P. yelkouan*),(*P. lherminieri*, *P. boydi*, *P. baroli*))	0.31	0.29	0.31	0.33	0.31
$\bullet$	Polytomy test $P$-value	$-$(0)	-(0.002)	0	0	0
	QS: (*A. grisea*, (*A. gravis*, *A. creatopus*, *A. carneipes*))	0.38	0.37	0.38	0.38	0.37
	QS: (*A. grisea*, *A. tenuirostris*)	0.38	0.39	0.35	0.36	0.35
	QS: (*A. tenuirostris*, (*A. gravis*, *A. creatopus*, *A. carneipes*))	0.24	0.24	0.27	0.26	0.28

### Divergence Dating Analysis

**Figure 5:**
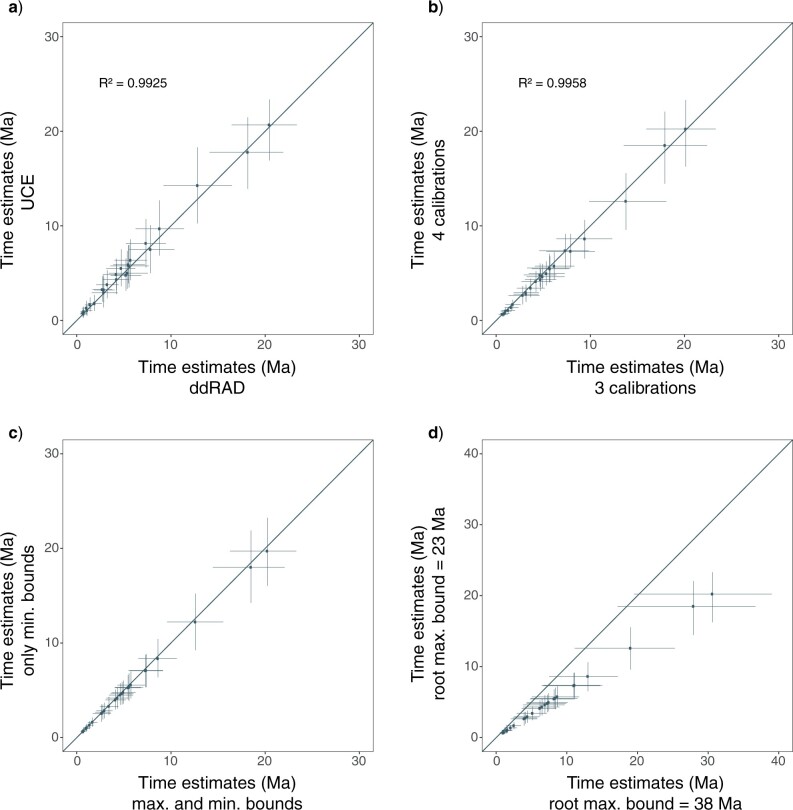
Effects of data type, calibration strategy, bound constraints and root maximum bounds. Posterior time estimates (points) and 95% credibility intervals (lines) using a) UCE versus PE-ddRAD data, b) four versus three calibration points, c) only minimum bounds versus minimum and maximum bounds, and d) setting the root maximum bound at 38 Ma versus 23 Ma.

For all sampled alignments except for one, the IR model had the highest posterior probability (Supplementary Table S4 available on Dryad). This model was interpreted as the best-fitting model and used in downstream MCMCTree analyses. Mean posterior time estimates using PE-ddRAD, UCE or TENT data sets differed only marginally (Fig. 5 and Supplementary Fig. S13 available on Dryad). Analyses using the TENT data set with two partitions produced posterior estimates with the narrowest credibility intervals while analyses using the UCE data set produced the largest credibility intervals. The slope of the regression line in the infinite-sites plot was highest for UCE data (0.51), was slightly lower for PE-ddRAD data (0.49), and dropped to 0.43 when using the TENT data set, meaning that 0.43 Ma of uncertainty was added to the 95% CI for every 1 Ma of divergence (Supplementary Fig. S14 available on Dryad). Except for the root, the points of the infinite-sites plot from the combined data set formed a straight line, indicating that the relatively high uncertainty in time estimates was mostly due to uncertainties in fossil calibrations (Rannala and Yang 2007).

Setting the root maximum bound to 38 Ma had the strongest impact on the divergence time estimates and resulted in more ancient estimates, with larger differences observed near the root. When we used constraints on minimum bounds, posterior estimates tended to be more recent and credibility intervals larger. Despite the fact that using four calibrations resulted in a slight truncation of the prior density on the age of the *Calonectris*–*Ardenna* node, mean posterior time estimates were nearly identical and variances were higher when only using three calibrations (Fig. 5 and Supplementary Fig. S13 available on Dryad).

### GC-biased Gene Conversion

Analyses of the relative site-frequency spectrum (SFS) for each mutation class showed a shift in the relative proportion of putative W-to-S and S-to-W mutations (Supplementary Fig. S15a available on Dryad). Putative W-to-S mutations were skewed towards high frequencies, and S-to-W mutations towards low frequencies, consistent with a prevalent gBGC-driven fixation bias (Bolívar et al. 2016). In addition, we observed that putative W-to-S mutations tended to maintain higher frequencies of the minor allele than S-to-W mutations, particularly at GC-rich areas (Supplementary Fig. S15b available on Dryad). In shearwaters, in contrast with the general trend in birds, species with smaller body mass (genus *Puffinus*) have smaller census sizes and are expected to have smaller effective population sizes, which should increase both the number of meioses per unit time and the efficacy of gBGC (Romiguier et al. 2010; Weber et al. 2014). Concordant with these expectations, we observed strong positive correlations between the overall proportion of putative W-to-S mutations and both the number of breeding pairs ($R^{2}= 0.552$ and $P = 1.5 \times 10^{-9})$ and the average body mass per taxon ($R^{2} = 0.731$ and $P = 1.1 \times 10^{-14}$; Supplementary Fig. S16 available on Dryad).

### Introgression Analyses

**Figure 6:**
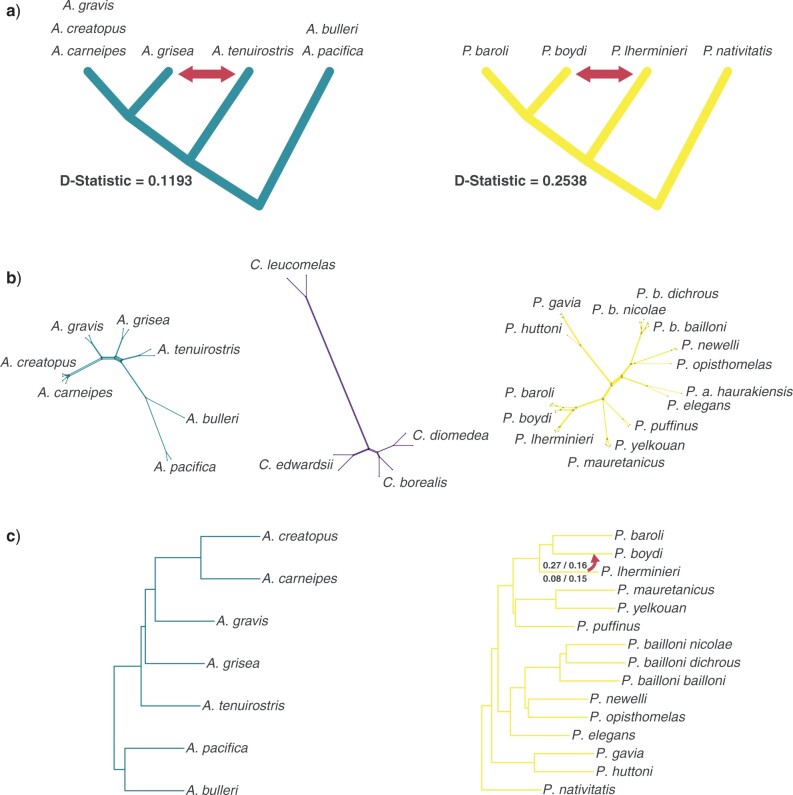
Introgression analyses in shearwaters. a) Gene-flow hypotheses obtained from D-statistic analyses (significant values) are shown with red arrows. Mean D-statistic values for the two cases are also shown. b) Neighbor-net networks for the three shearwater genera. c) Maximum pseudolikelihood SNaQ networks for *Ardenna* (h $= 0$) and *Puffinus* (h $= 1$). For the inferred hybridization event in *Puffinus*, optimized inheritance probabilities for the minor hybrid edge ($\gamma )$ using PE-ddRAD/UCE gene trees (above) and PE-ddRAD SNPs/UCE SNPs (below) are shown.

D-statistic tests found clear evidence for an excess of shared derived alleles consistent with introgression between *A. grisea* and *A. tenuirostris* (D-statistic $= 0.1193$) and even stronger evidence between *P. boydi* and *P. lherminieri* (D-statistic $= 0.2538$) (Fig. 6a). For these two potential cases of introgression, shared ancestral variation accounted for 20–31% of shared derived alleles in SNPs with ABBA pattern (Supplementary Table S5 available on Dryad). Loci with ABBA SNPs were not significantly more variable than average loci, although they were in the upper part of the distribution for the *P. boydi*–*P. lherminieri* case. In the *A. tenuirostris*–*A. grisea* case, we found a significantly higher proportion of ABBA patterns generated by putative W-to-S mutations than expected by chance ($P$-value $= 0.0257$), which might indicate a role of gBGC in generating these patterns. Finally, we observed that potentially introgressed species had the highest individual heterozygosities (Supplementary Fig. S17 available on Dryad).

Neighbor-net networks for each genus showed low levels of reticulation and were consistent with concatenated and coalescent-based phylogenetic analyses (Fig. 6b). However, we observed reticulation in areas where D-statistics showed evidence for an excess of shared derived alleles between non-sister taxa.

The TICR test detected a significant excess of outlier quartets in the data for *Puffinus*, when using PE-ddRAD or UCE gene tree data, suggesting that the coalescent tree inferred without introgression did not adequately fit the data. We recovered a deficit of high concordance factors (CF) and an excess of low CF (Supplementary Fig. S18 available on Dryad). Nonetheless, the observed values at the extremes tended towards the expected values when increasing the BS threshold for collapsing branches in the gene trees. When branches with BS $<$ 50 were collapsed, the TICR test was no longer significant, suggesting that the excess of outlier quartets was caused by including noise in form of inaccurate gene tree branches. Using a program like BUCKy (Larget et al. 2010) to calculate the CF from gene trees can provide an advantage, since it also considers uncertainty in gene tree estimation. It is noteworthy that in all PE-ddRAD data sets (but not in UCE data sets), *P. boydi* and *P. lherminieri* were found more frequently in four-taxon sets that did not fit the tree model with ILS compared to any other taxon. The TICR test for *Ardenna* was not significant and all four-taxon sets fitted the tree model with ILS.

Consistent with the TICR test results, we detected a lack of sharp improvement in the slope heuristic in both *Puffinus* and *Ardenna* data sets. Nonetheless, we detected a general sharper decrease up to h $= 2$ in *Puffinus*, although inferred reticulation events had $\gamma < 5$%. The only introgression event with $\gamma > 5$% was from *P. lherminieri* to *P. boydi*. For candidate networks assuming introgression between *P. lherminieri* and *P. boydi*, log pseudolikelihood values obtained from PE-ddRAD data were either lower than or similar to those obtained in optimized networks with h $= 1$, showing support for introgression between these taxa (Supplementary Table S6 available on Dryad). The directionality of introgression could not be determined reliably because optimization of candidate networks showed very similar log pseudolikelihood values for pairs of candidate networks with reversed direction of introgression. However, inheritance probabilities were much higher when assuming introgression from *P. lherminieri* to *P. boydi* than when assuming the opposite (Fig. 6c and Supplementary Table S6 available on Dryad).

## Discussion

Our results demonstrate the power of integrating UCE and RAD markers for resolving the phylogenetic relationships of a group of pelagic seabirds characterized by rapid diversification events that have confounded previous phylogenetic studies. To our knowledge, our study is the first to compare and integrate UCE and paired-end ddRAD data sets in a phylogenomic context using comparably phased sequence alignments for both data sets. Here, we propose a strategy to optimize RAD-Seq data for phylogenetic analyses, we consider aspects of our methodological approach that may be of help to future studies, we discuss case-by-case how the integrative use of PE-ddRAD-Seq and UCE data with phylogenetic and introgression analyses allows identification of the causes of phylogenetic discordance, and we discuss the systematic implications of our phylogenetic results.

### RAD-Seq Data Set Optimization for Phylogenetic Analyses

There are two main factors that affect the number of orthologous loci recovered in RAD-seq data sets: 1) divergence times between lineages and 2) filtering and assembly parameters applied to orthology inference. Considerable research attention has focused on exploring how the number of orthologous loci decreases with increasing divergence times (Rubin et al. 2012; Cariou et al. 2013) and how filters based on taxon coverage affect data set size and the ability to resolve phylogenetic relationships (Wagner et al. 2013; Leaché et al. 2015; Díaz-Arce et al. 2016; Tripp et al. 2017). However, phylogenomic studies have generally neglected the optimization of assembly parameters in order to minimize the inclusion of paralogous or repetitive loci (but see Hosegood et al. 2020), a procedure that is common practice in population genomics analyses (Paris et al. 2017; Rochette and Catchen 2017). To fill this gap, we used a two-step optimization process to assess the impact of common issues in RAD-Seq, such as sequencing error and paralog content, on phylogenetic reconstruction. However, our analyses revealed a minor effect of assembly parameters on phylogenetic reconstruction, compared to taxon coverage. Our study adds to previous evidence showing a major effect of taxon coverage on phylogenetic reconstruction when using RAD-Seq data sets (Díaz-Arce et al. 2016; Tripp et al. 2017). We recommend that phylogenetic studies using RAD-Seq data should explore several taxon coverage filters in order to maximize the phylogenetic informativeness of their data sets.

### Considerations on Methodological Approaches for Phylogenetic Inference

As expected, differences in methodological approaches can severely affect the phylogenetic inference. First, in cases of extreme levels of ILS, concatenation analyses may result in an average topology that differs from the true species tree (Mendes and Hahn 2018). On the other hand, low phylogenetic information per locus in PE-ddRAD and UCE data sets might result in poorly resolved gene trees, and in such cases, concatenation methods can be more accurate than summary coalescent approaches (Mirarab et al. 2016; Springer and Gatesy 2016). In our case, concatenation and summary coalescent approaches produced largely congruent topologies that expressed a lower degree of incongruence than analyses using different data sets and different levels of missing data. Nevertheless, concatenation exacerbated systematic error in two cases, leading to high confidence in alternative relationships in areas of elevated ILS (Fig. 4).

Second, Andermann et al. (2018) found that using IUPAC consensus sequences performed better than using contig sequences for estimating the tree topology of a recently diverged group of *Topaza* hummingbirds under the MSC model. Our concatenation analyses using UCE contig alignments produced topologies nearly identical to those from analyses using the IUPAC consensus sequence alignments. However, consistent with Andermann et al. (2018), we observed a tendency towards a poorer performance of contig sequences at recovering the monophyly of species and subspecies (Supplementary Table S3 available on Dryad). We evidenced that using IUPAC sequence alignments resulted in a reduction of mean locus length ($\sim $100 bp shorter). This was due to the inability of accurate phasing towards the extremes of UCEs because of lower read coverages (we required a minimum of 5 reads per haplotype to include a position). This approach is more conservative than using the full contig sequence and therefore results in a reduction of the number of PIS. However, it delivers a higher reliability in base calling and a true representation of polymorphism that can be particularly useful at recent timescales. Researchers working with UCE data face a trade-off between longer alignments with higher amounts of PIS and more reliable base calling allowing the inclusion of polymorphism data.

Third, partitioning strategy can affect the outcome of phylogenetic analyses. For example, maximum likelihood, when used to analyses an unpartitioned concatenated alignment from different loci, can converge to a tree other than the species tree as the number of loci increases (Roch and Steel 2015). Our concatenation analyses using the partitioned UCE 75 data set recovered exactly the same topology compared to unpartitioned analyses, with only slight changes in branch support, showing that the topology was relatively insensitive to the partitioning scheme used. This was likely due to a smaller effect of evolutionary rate heterogeneity among loci at shallow phylogenetic scales like the one we were working than at deep timescales.

Fourth, increasing the number of individuals strongly improves species tree estimation in ASTRAL-III when branch lengths are extremely short (Rabiee et al. 2019). We found evidence for this pattern in our UCE ASTRAL-III trees, where we found incongruences affecting nodes where individuals had been removed to avoid the inclusion of fragmentary sequences. We also observed an improvement in precision when analyses were performed after contracting very low support branches (BS $< 10$) in the gene trees, as previously suggested by Zhang et al. (2017) (Supplementary Table S3 available on Dryad).

### Divergence Dating with UCE and PE-ddRAD

Collins and Hrbek (2018) showed consistent discrepancies in divergence time estimates using different phylogenomic markers under strict and relaxed clock models. The discrepancies were explained by temporal differences in phylogenetic informativeness (PI). However, their analyses showed that UCE and ddRAD data sets showed similar temporal patterns of PI and resulted in similar divergence time estimates. Our analyses using UCE and PE-ddRAD empirical data sets also showed very similar estimates. However, UCE estimates tended to be slightly older and have wider credibility intervals than PE-ddRAD estimates probably due to UCE data sets having lower PI at the timescales covered in this study.

Theoretically, for infinitely long alignments, an infinite-sites plot, which measures the uncertainty in the divergence time posterior (width of the credibility interval vs. posterior mean of node ages) should converge onto a straight line (Rannala and Yang 2007). The slope of this line represents the amount of uncertainty in time estimates per 1 myr of divergence solely due to uncertainties in the fossil calibrations. The points of the infinite-sites plot from the UCE and the PE-ddRAD data sets did not form a straight line, suggesting that uncertainties in time estimates were due both to limited data as well as uncertainties in the fossil calibrations. On the other hand, the points from the TENT data set formed a straight line (Supplementary Fig. S14 available on Dryad), indicating that the relatively high uncertainty in time estimates was mostly due to uncertainties in fossil calibrations and that increasing the amount of data would only marginally reduce the credibility intervals (Rannala and Yang 2007). We thus show that combining both data sets resulted in lower uncertainties in divergence time estimates.

### Integrative Approach using UCE and PE-ddRAD to Disentangle Phylogenetic Discordance

The utility of RAD-seq and target capture approaches for phylogenetic estimation across different timescales has been widely demonstrated (Faircloth et al. 2012; Cruaud et al. 2014; Smith et al. 2014; McCluskey and Postlethwait 2015). However, only a handful of studies have explored the utility of both approaches in phylogenetic studies (Leaché et al. 2015; Harvey et al. 2016; Manthey et al. 2016; Collins and Hrbek 2018). Using a higher number of taxa and loci than these previous studies, we show the advantages of integrating PE-ddRAD-Seq and UCE data to infer the phylogenetic relationships of a challenging group, the shearwaters, across a range of timescales using concatenation and coalescent approaches.

Despite finding only minor data-type effects, data sets from different markers and levels of missing data tended to better resolve short internodes at different timescales (Fig. 4 and Supplementary Fig. S5 available on Dryad). For instance, the UCE 95 data set, which contained the lowest number of PIS, showed the highest support across methods (with the exception of SVDQuartets) for the sister relationship between *Ardenna* and *Calonectris* (18.5 Ma), but showed the poorest performance at recovering the monophyly of recently diverged taxa (Fig. 4). This shows that the phylogenetic signal of this data set is stronger near the root and weaker at shallow timescales. Conversely, the data set with the highest number of PIS (PE-ddRAD 75) recovered alternative topologies (with low support) for the relationships between the three genera, but recovered the monophyly of all the species (and subspecies). Finally, phylogenetic analyses using the TENT 75 matrix performed well at both deep and shallow timescales, showing the advantages of combining different data types in a single analysis. Our study is consistent with previous findings that combining data types leads to higher resolution on short internodes (Jarvis et al. 2014), and it is noteworthy that, due to high rate heterogeneity between PE-ddRAD loci and UCEs, the observed data-type effects could also reflect poor model fit (Reddy et al. 2017).

Despite the strong support in most of our phylogeny, we found phylogenomic conflict associated with short internodes in three areas of the tree (Degnan and Rosenberg 2006). Short internode lengths are usually associated with topological conflict. This conflict arises because short speciation intervals 1) accumulate few substitutions, resulting in a low number of informative sites and, 2) they increase the probability of finding different gene histories due to ILS (Alda et al. 2019).

The first area of conflict was the short internode near the root separating *Ardenna* and *Calonectris* from *Puffinus* (triangle in Fig. 3). When using the PE-ddRAD data set with the highest number of PIS, a sister relationship between *Ardenna* and *Puffinus* received the highest quartet support in ASTRAL-III analyses. The remaining data sets provided the highest quartet support for a sister relationship between *Ardenna* and *Calonectris* and the support for this arrangement decreased when increasing the number of PIS in the data set (Tables 1 and 2). These results show how high levels of ILS resulted in different phylogenetic signals across data sets and could indicate that our most variable data set is affected by homoplasy due to saturation at this timescale. In addition, the increased support for the *Ardenna* $+$  *Puffinus* clade compared to the alternative topology in PE-ddRAD data sets could also indicate molecular convergence between *Ardenna* and *Puffinus*, which show similarities in morphology and diving behavior and were historically placed in the same genus (Penhallurick et al. 2004).

The split of the three North Atlantic lineages of *Puffinus* (1: *P. lherminieri*, *P. baroli* and *P. boydi*, 2: *P. puffinus*, and 3: *P. mauretanicus* and *P. yelkouan*) (Fig. 3a) represents the second conflict associated with a short internode. In this case, our analyses largely supported a sister relationship between *P. puffinus* and the Mediterranean clade. Nonetheless, only the PE-ddRAD 75 and the TENT 75 data sets clearly rejected the polytomy test (Table 2). When two speciation events occur at the same time (hard polytomy) and gene tree heterogeneity is mostly caused by ILS, the multispecies coalescent model (MSC) predicts equal frequencies for each of the three topologically informative unrooted quartet topologies defined around the short internode (Degnan and Rosenberg 2009). Our data show that these frequencies are nearly equal for most analyses (Table 2). In addition, our introgression analyses confirmed that no introgression had occurred between *P. puffinus* and the *P. boydi, P. baroli*, and *P. lherminieri* lineage. These observations suggest a nearly simultaneous divergence of the three lineages that resulted in high levels of ILS.

The last short internode-associated phylogenetic conflict was the relationship between the two all-dark colored *Ardenna* species (*A. tenuirostris* and *A. grisea*). Concatenation analyses showed strong support for the sister relationship between *A. grisea* and the *A. gravis, A. creatopus*, and *A. carneipes* clade across data type and levels of missing data (Fig. 4). On the other hand, the alternative topology of a sister relationship between the two all-dark species received ASTRAL-III quartet supports that were nearly as high as (PE-ddRAD data) or higher (UCE data) than the main topology, and at least 8% higher than the remaining alternative topology. These results, together with significant D-statistic values between *A. grisea* and *A. tenuirostris* (Fig. 6), suggest that introgression could have generated this pattern. However, our PhyloNetworks analysis did not support introgression between these two species (Supplementary Table S6 available on Dryad). Moreover, our evaluation of SNPs with strong signals of introgression suggested a role of GC-biased gene conversion (gBGC) in generating these patterns (Supplementary Table S5 available on Dryad). Interestingly, *A. tenuirostris* and *A. grisea* have two of the highest population sizes amongst all shearwaters and thus a likely increased efficacy of gBGC (Weber et al. 2014). Mutation rate variation among different lineages can also lead D-statistics to incorrectly infer introgression (Blair and Ané 2019). Our clock model selection analysis recovered the independent rates clock as the best-fit model, showing important rate heterogeneity among the shearwaters (Supplementary Table S4 available on Dryad). Branch lengths in phylogenetic analyses showed that rate heterogeneity is particularly high in the genus *Ardenna* (Fig. 3a). In line with these observations, we recovered lower D-statistic values between *A. grisea* and *A. tenuirostris* when we used *A. creatopus* or *A. carneipes* (longer branches) as a sister group to *A. grisea* than when we used *A. gravis* (shorter branch). Furthermore, the shearwater ancestor was likely all dark in coloration, because most species of *Procellaria*, the sister genus to the shearwaters (Estandia 2019), are all dark. Consequently, *A. grisea* and *A. tenuirostris* may share ancestral variation that could also produce a signature of shared ancestry (Smith and Kronforst 2013). These two species also have large differences in range size which could result in D-statistics being misled by ancestral population structure. Taking all these observations into consideration, phylogenetic conflict in this case was likely driven by ILS in combination with rate heterogeneity, gBGC, shared ancestral variation, and ancestral population structure.

Our results allow us to conclude that integrating different types of markers together with phylogenetic and introgression analyses provides a better understanding of the causes of phylogenetic incongruence and can be particularly useful for interpreting phylogenetic conflict at short internodes.

### Phylogeny of the Shearwaters

Previous phylogenies inferred using mtDNA provided poor support for the relationships among the major shearwater lineages (Austin 1996; Heidrich et al. 1998; Nunn and Stanley 1998; Austin et al. 2004). Historically, *Ardenna* species were included within *Puffinus* based on their morphology, osteology and behavior. We obtained full support for the monophyly of the three shearwater genera. We also recovered *Calonectris* and *Ardenna* as sister lineages (Fig. 3a), a novel arrangement, which differs from previous phylogenies. However, the internode of the clade which included *Ardenna* and *Calonectris* was very short, suggesting the succession of two rapid splits that gave rise to the three extant genera. The fossil record for the Procellariiformes is not rich in well-resolved older taxa, but it does document approximately simultaneous primary records of the three shearwater genera stem lineages in the early to middle Miocene ($\sim $14–15.2 Ma) (Miller 1961; Olson 2009), supporting our results.

In the genus *Calonectris*, the short internode of the *C. borealis*–*C. diomedea* clade and its high levels of ILS suggest that the speciation events between the North Atlantic species occurred over a short period of time. Using mtDNA data, Gómez-Díaz et al. (2006) recovered *C. borealis* and *C. edwardsii* as sister species. D-statistic analyses showed that probably no introgression occurred between *C. edwardsii* and *C. borealis*, suggesting that phylogenetic discordance was likely caused by ILS alone.

Some lineages of *Ardenna* exhibit strong morphological stasis. For instance, fossils of the extinct *A. conradi* from the middle Miocene were very similar to the extant *A. gravis* (Wetmore 1926). Morphological stasis and similar diving adaptations may explain the resemblance of *A. grisea* and *A. tenuirostris* to *P. nativitatis*, and likely caused the previous placement of these species together under the polyphyletic group *Neonectris* (Kuroda 1954). Phylogenetic analyses based on mtDNA found the *Neonectris* group to be polyphyletic (Austin 1996; Nunn and Stanley 1998; Pyle et al. 2011). We also recovered this polyphyly and confirmed the phylogenetic relationships among *Ardenna* species recovered previously (Pyle et al. 2011).

We recovered *P. nativitatis* and the New Zealand species *P. gavia* and *P. huttoni* as the first two splits within *Puffinus*, in agreement with previous studies (Austin et al. 2004; Pyle et al. 2011). Relationships among the remaining species of *Puffinus* were previously unresolved. We recovered fully resolved relationships among these species, which have revealed consistent biogeographic patterns. One of the most relevant results is the polyphyly of the small-sized shearwaters of the *P. assimilis*–*lherminieri* complex, which were historically placed together based on their body size (Austin et al. 2004) (Fig. 3a). Changes in body size are frequent in the evolutionary history of Procellariiformes (Nunn and Stanley 1998). Our results suggest that, on a smaller scale, changes in body size are also common along the shearwater phylogeny and may represent an important trait in the diversification process of pelagic seabirds.

### Introgression between P. boydi and P. lherminieri

Despite strong philopatry to breeding colonies and, in most cases, a lack of overlap between breeding areas of closely related species, hybridization has been documented between several sibling species of Procellariiformes, including shearwaters (Genovart et al. 2012; Booth Jones et al. 2017; Masello et al. 2019). However, the occurrence of ancestral introgression in Procellariiformes has not been studied. Our D-statistic tests found an excess of shared derived alleles between *P. boydi* and *P. lherminieri* (Fig. 6). Despite full support for the sister relationship between *P. boydi* and *P. baroli* in all phylogenetic analyses, 28% of the gene trees supported a sister relationship between *P. boydi* and *P. lherminieri*, and 18% supported a sister relationship between *P. baroli* and *P. lherminieri*. Additionally, the NeighbourNet network showed smaller genetic distances between *P. boydi* and *P. lherminieri* than between *P. baroli* and *P. lherminieri*. Because D-statistic tests can be misled by factors such as ancestral population structure (Eriksson and Manica 2012) or low Ne (Martin et al. 2015), we used a phylogenetic network approach to simultaneously account for ILS and gene flow in *Puffinus.* We also evaluated the need to account for gene flow using the TICR test. Although the TICR test showed that a tree model with ILS could explain the observed concordance factors (Supplementary Fig. S18 available on Dryad), candidate networks using PE-ddRAD data showed support for introgression between these taxa (Supplementary Table S6 available on Dryad).

The inference of the directionality of introgression using phylogenetic network approaches can be challenging. For instance, SNaQ assumes that no two edges can be part of the same cycle (Solís-Lemus and Ané 2016). This assumption precludes the possibility of inferring gene flow between two taxa in both directions in the same analysis. In addition, gene tree distributions from these alternative topologies may be indistinguishable (Pardi and Scornavacca 2015). However, the network with *P. boydi* as the recipient of genetic material from *P. lherminieri* had much higher inheritance probabilities than those with *P. lherminieri* as recipient. These facts, together with increased heterozygosity in *P. boydi* compared to *P. lherminieri* and *P. baroli*, point to *P. boydi* as the recipient of genetic material.

Optimized inheritance probabilities on the fixed network with gene flow from *P. lherminieri* to *P. boydi* were lower when using UCE data than when using PE-ddRAD data. Ultraconserved elements are likely under strong purifying selection (Bejerano et al. 2004; Harvey et al. 2016) and thus introgressed alleles may be more rapidly removed in these areas of the genome (Juric et al. 2016). In concordance with our results, although some introgressed alleles can be adaptive (Hedrick 2013), selection is primarily known to act against introgressed DNA (Sankararaman et al. 2014), particularly in regulatory regions (Petr et al. 2019) where UCEs are usually located (Bejerano et al. 2004).

The fossil record has documented the presence of both *P. boydi* and *P. lherminieri* in Bermuda during the Pleistocene, where *P. boydi* probably outcompeted *P. lherminieri* until it was extirpated, evidently due to human-introduced predators (Olson 2010). Thus, these two species may have hybridized during the Pleistocene. In addition, *P. boydi* and *P. lherminieri* show some contemporary overlap in their wintering areas (Ramos et al. 2020), which may be a relic of a previously higher overlap in distribution. Future studies should use population genomics data in order to confirm whether the observed patterns are due to ancestral introgression between these two species or to ancestral population structure.

## Conclusions

Here, we demonstrate the power of integrating UCE and RAD markers, and employing state-of-the-art phylogenetic and introgression analyses, for resolving phylogenetic discordances associated with short internodes. We show that using markers that evolve at different rates allows a detailed exploration of the causes of phylogenetic discordance at different timescales. Our approach provides power to fully resolve complex evolutionary scenarios, such as rapid radiations and introgression histories. Applied to the shearwater problem, our phylogenetic results identified novel relationships and resolved the rapid radiation in the genus *Puffinus*. We have demonstrated that most phylogenetic discordance in shearwaters is driven by high levels of ILS due to rapid speciation events. However, we found evidence for ancestral introgression between *P. boydi* and *P. lherminieri*.

## Data Accessibility

All raw sequence data are archived on the European Nucleotide Archive (ENA) under the accession number PRJEB38458. Scripts used in this project are in the GitHub repository (https://github.com/jferrerobiol/shearwater_phylogenomics).

## Supplementary Material

Data available from the Dryad Digital Repository: http://dx.doi.org/10.5061/dryad.d51c5b00t.
